# Design of Smartphone-Assisted Point-of-Care Platform for Colorimetric Sensing of Uric Acid via Visible Light-Induced Oxidase-Like Activity of Covalent Organic Framework

**DOI:** 10.3390/s23083881

**Published:** 2023-04-11

**Authors:** Qi Kang, Yulong Xu, Xuwei Chen

**Affiliations:** College of Sciences, Northeastern University, Shenyang 110819, China

**Keywords:** covalent organic framework, oxidase-like activity, colorimetric sensing, smartphone platform, uric acid

## Abstract

Monitoring of uric acid (UA) levels in biological samples is of great significance for human health, while the development of a simple and effective method for the precise determination of UA content is still challenging. In the present study, a two-dimensional (2D) imine-linked crystalline pyridine-based covalent organic framework (TpBpy COF) was synthesized using 2,4,6-triformylphloroglucinol (Tp) and [2,2′-bipyridine]-5,5′-diamine (Bpy) as precursors via Schiff-base condensation reactions and was characterized with scanning electron microscopy (SEM), Energy dispersive X-ray spectroscopy (EDS), Powder X-ray diffraction (PXRD), Fourier transform infrared (FT-IR) spectroscopy, and Brunauer–Emmett–Teller (BET) assays. The as-synthesized TpBpy COF exhibited excellent visible light-induced oxidase-like activity, ascribed to the generation of superoxide radicals (O_2_^•−^) by photo-generated electron transfer. TpBpy COF could efficiently oxidase the colorless substrate 3,3′,5,5′-tetramethylbenzydine (TMB) into blue oxidized TMB (oxTMB) under visible light irradiation. Based on the color fade of the TpBpy COF + TMB system by UA, a colorimetric procedure was developed for UA determination with a detection limit of 1.7 μmol L^−1^. Moreover, a smartphone-based sensing platform was also constructed for instrument-free and on-site detection of UA with a sensitive detection limit of 3.1 μmol L^−1^. The developed sensing system was adopted for UA determination in human urine and serum samples with satisfactory recoveries (96.6–107.8%), suggesting the potential practical application of the TpBpy COF-based sensor for UA detection in biological samples.

## 1. Introduction

Uric acid (UA) is the final oxidized product of purine metabolism in the human body [1]. Generally, UA usually exists in human urine and serum, and the normal concentration of UA is 1.4–4.5 mmol L^−1^ in urine and 0.12–0.46 mmol L^−1^ in serum [2]. The abnormal level of UA is usually a sign of some diseases, such as gout [3], renal disease [4], urolithiasis [5], and Lesh–Nyhan syndrome [6], and therefore the monitoring of UA contents in biological samples is of great significance to human health.

Up until now, various techniques have been developed for UA detection, including high-performance liquid chromatography (HPLC) [7], fluorescence spectroscopy [8], chemiluminescence [9], electrochemical techniques [10,11], etc. Typically, these detection processes usually adopt the dual natural enzyme reaction system based on uricase and horseradish peroxidase (HRP) [12], though most of these techniques are merited with high detection sensitivity, while intricate sample preparation and expensive instrumentations are usually needed to accomplish the detection (Table 1). Moreover, the poor stability and severe reaction condition of the catalysts adopted in these detection processes further restrict their practical applications. Recently, colorimetric detection technique based on oxidase-mimic enzymes are gaining increasing attention due to the advantages of simplicity, rapidity, sensitivity, and low cost, and various materials, such as Q-graphene [13], metal–organic frameworks [14], noble metal nanoparticles [15], and alloy nanoparticles [16], have been used as artificial enzymes or enzymes mimetic for sensitive UA detection, attributed by their high efficiency of catalysis and kinetics similar to natural enzymes. Tarasankar et al. reported the colorimetric determination of UA with hierarchical porous Ni–MnO_2_ nanoparticles based on the redox reaction between UA and oxTMB [17], which displayed a quite low detection limit. Wang et al. designed the MOF-based Pt nanoparticles (PtNPs@ZIF) with excellent peroxidase-like activity towards substrate TMB due to its unique electron transfer mechanism [18] and applied it to UA detection (as UA could suppress the oxidation of TMB). Liu et al. prepared core-shell silver-carbon dots (Ag-CDs) and nanocomposites with excellent peroxidase-like and surface-enhanced Raman scattering (SERS) activities [19], achieving the highly sensitive UA detection via the synergistic colorimetric reaction and enhanced SERS technique. 

Covalent organic frameworks (COFs) as metal-free nanoenzymes have gained increasing attention for catalytic applications due to their excellent chemical stability, large surface area, high porosity, and tunable functionality that is superior to other porous materials. It is worth mentioning that some COF materials exhibit promising light-activated properties due to the fact that their C, N-rich skeleton structure endows them with the unique π-π array frameworks [20,21]. Lin et al. prepared two donor−acceptor heterosporous COFs (ETTA-Tz COF and ETTA-Td COF) by using 4,4′-(thiazolo [5,4-d]thiazole- 2,5-diyl)dibenzaldehyde (Tz) or 4,4′-(benzo[c] [1,2,5]thiadiazole-4,7-diyl)dibenzaldehyde (Td) as precursors [22], and the narrow band gap and efficiently charge transport along the COF skeleton offered the as-prepared ETTA-Tz/ETTA-Td COF with excellent oxidase-like activity under light irradiation. Xu et al. reported a two-dimensional COF (TAS-COF) prepared by 2,4,6-triformylphloroglucinol (Tp) and 3,7-diaminodibenzo[b,d] thiophene sulfone (DAS), which owned excellent photocatalytic performance under visible light irradiation and was applied in the accurate determination of UO_2_^2+^ content [23].

Recently, biosensors based on smartphone platforms have displayed great potential in the fields of healthcare monitoring [24,25,26], which is ascribed to the merits of portability, low cost, and easy operation. A smartphone can also be performed as an essential colorimetric detecting platform for field inspection, and the development of a smartphone-based platform for UA detection is worthy of consideration.

**Scheme 1:**
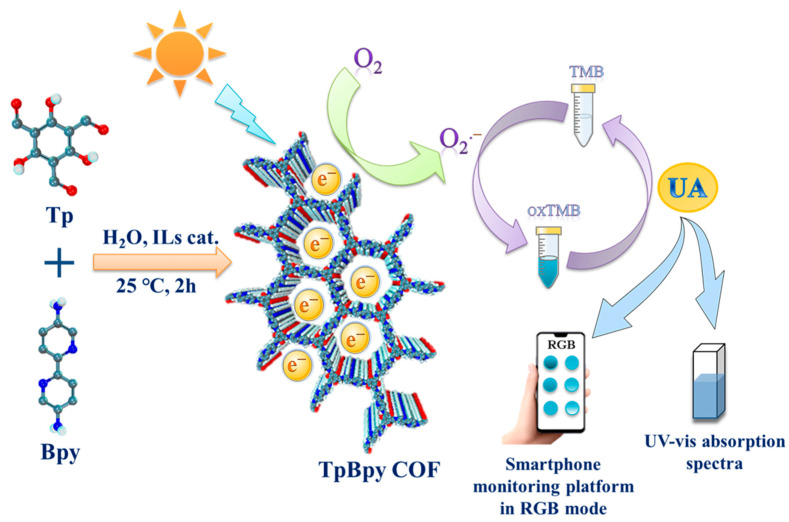
The schematic diagram of UA detection based on the light-responsive TpBpy COF.

In the present study, a two-dimensional COF (TpBpy COF) with excellent photocatalytic performance was designed and synthesized via the Schiff-base condensation reaction of *C*_3_-symmetric 2,4,6-triformylphloroglucinol (Tp) and *C*_2_-symmetric [2,2′-bipyridine]-5,5′-diamine (Bpy) using ionic liquid as catalyst (Scheme 1). The as-synthesized TpBpy COF exhibited favorable oxidase-like activity under visible light irradiation, which could generate O_2_^•−^ via photo-generated electron transfer. The generated O_2_^•−^ could directly catalyze the oxidization of colorless TMB into blue oxidized TMB (oxTMB). The redox reaction between UA and oxidized TMB [27] induced obvious color fading of the TpBpy COF + TMB system, and thus a COF-based colorimetric sensor and a smartphone-based platform were developed for the sensitive sensing of UA content. The TpBpy COF-based colorimetric sensor and smartphone-based platform demonstrated their practicability by the accurate determination of UA contents in human serum and urine samples.

## 2. Materials and Methods

### 2.1. Chemicals and Reagents

2,4,6-triformylphloroglucinol (Tp) and [2,2′-bipyridine]-5,5′-diamine (Bpy) were purchased from Alpha Chemical Co., Ltd. (Zhengzhou, China). 3,3,5,5-tetram [2,2′-bipyridine]-5,5′-diamine ethylbenzydine (TMB), o-Phenylenediamine (OPD), and 2,2′-azino-bis(3-ethylbenzothiazoline-6-sulfonic acid) (ABTS) were provided by Aladdin Industrial Inc. (Shanghai, China). Uric acid (UA), HAc, and NaAc were obtained from Sinopharm Chemical Reagent (Shanghai, China). Ionic liquids [BmimN(CN)_2_] were acquired from Chengjie Chemical Co., Ltd. (Shanghai, China).

Unless otherwise stated, all chemicals used were at least analytically pure and used without any purification. Notably, 18 MΩ cm deionized water was used in all experiments.

### 2.2. Synthesis of TpBpy COF

TpBpy COF was synthesized according to a reported procedure with some modifications [28]. Briefly, 0.08 mmol of Tp, 0.12 mmol of Bpy, and 100 μL of [BmimN(CN)_2_] were dissolved in 2.0 mL H_2_O, and, then, the mixture was stirred at room temperature for 2 h. Thereafter, the produced red solid was collected through centrifugation and was washed three times with acetone and ethanol, respectively. Finally, the product was dried under a vacuum at 60 °C for 12 h.

### 2.3. Characterization of TpBpy COF

Powder X-ray diffraction (PXRD) was collected on a Maxima XRD-7000 diffractometer (Shimadzu, Kyoto, Japan). N_2_ adsorption-desorption isotherm was obtained by an Autosorb-IQ-MP-C automatic gas adsorption analyzer at 77 K (Quantachrome, Boynton Beach, FL, USA). Scanning electron microscopy (SEM) images were collected on a SU8010 field-emission electron microscope at a voltage of 5.0 kV (Hitachi, Tokyo, Japan). Fourier transform infrared (FT-IR) spectra were recorded using a Nicolet-6700 FT-IR spectrophotometer (Thermo Scientific, Waltham, MA, USA). Ultraviolet-visible (UV-vis) absorption spectra were obtained on a U-3900 UV-Vis spectrophotometer (Hitachi, Tokyo, Japan). Visible light was generated by a white-light LED lamp (λ ≥ 420 nm, power density = 62 mW cm^−2^, Guanyu Lighting Co., Ltd., Guangzhou, China).

### 2.4. Steady-State Kinetic Studies of TpBpy COF as Oxidase-Mimic

The steady-state kinetic studies were carried out by changing the concentration of TMB under fixed TpBpy COF levels. In brief, 100 μL TMB of different concentrations and 50 μL COF solution (0.1 mg mL^−1^) were mixed with 850 μL HAc-NaAc buffer (0.2 mol L^−1^, pH 4.0). After irradiation with white-light LED lamp for 15 min, the absorbance at 652 nm was recorded.

### 2.5. Colorimetric Detection of UA

Typically, 50 μL COF solution (0.1 mg mL^−1^) and 250 μL TMB solution (1.0 mmol L^−1^) were mixed with 600 μL HAc-NaAc buffer (0.2 mol L^−1^, pH 4.0), then 100 μL UA solution (prepared in 0.1 mol L^−1^ PBS buffer, pH 7.4) was added into the mixture subsequently, and the final volume of the detection system was 1 mL. After that, the resultant mixture was irradiated under a white-light LED lamp (λ ≥ 420 nm, 62 mW cm^−2^) for 15 min to stimulate the catalytic reaction. The absorbance of this system at 652 nm was recorded. The absorbance difference A_0_ − A (A_0_ and A represent the absorbance at 652 nm in the absence/presence of UA) was adopted to fabricate the calibration curve.

### 2.6. Detection of UA by the Smartphone Mode

In an EP tube, 50 μL COF solution (0.1 mg mL^−1^), 250 μL TMB solution (1.0 mmol L^−1^), 100 μL UA solution (with different concentrations, 0.05–0.8 mmol L^−1^), 600 μL HAc-NaAc buffer (0.2 mol L^−1^, pH 4.0) were added and irradiated under a white-light LED lamp (λ ≥ 420 nm, 62 mW cm^−2^) for 15 min. Thereafter, removing 100 μL of the resultant mixture into a cover of centrifugal pipe. Digital images were collected in a self-made box (19.5 cm × 18.5 cm × 16.5 cm) by a Mi 10 smartphone under a white-light LED lamp (λ ≥ 420 nm, 62 mW cm^−2^) through the “Color Grab” app and processed into R, G and B parameters. The distance between the smartphone’s camera and the tested sample was 20 cm. The usage of LED lamp as the visible light source was due to its stable and intensive light irradiation.

### 2.7. Determination of UA Content in Real Samples

Human blood samples were provided by the Hospital of Northeastern with the volunteers’ consent. The blood samples were dealt with refrigerating centrifugating (10,000 rpm) for 20 min, and then the collected supernatant was diluted 5-fold with 0.1 mol L^−1^ PBS buffer (pH 7.4).

Urine sample was donated by the author. The urine samples are 10-fold, diluted with 0.1 mol L^−1^ PBS buffer (pH 7.4), and then directed to colorimetric detection.

## 3. Results and Discussion

### 3.1. Synthesis and Characterizations of TpBpy COF

**Figure 1:**
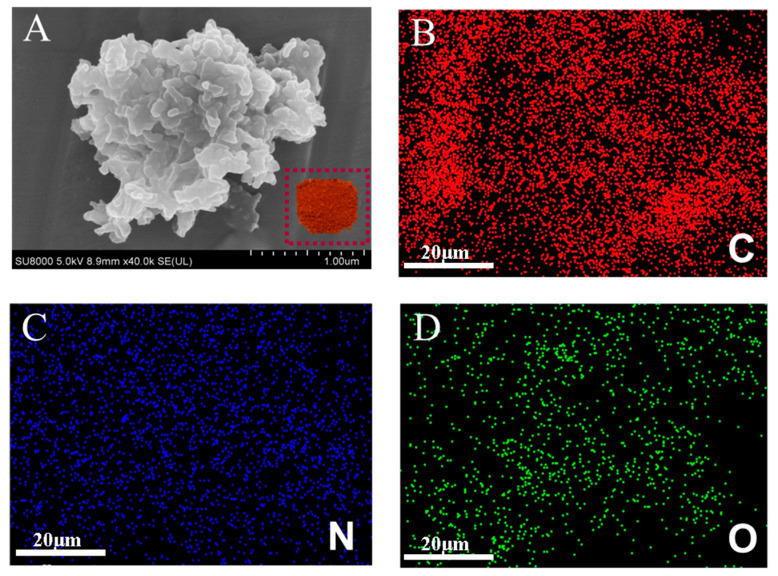
(**A**) SEM image of TpBpy COF (Insert: the picture of TpBpy COF). (**B**–**D**) EDS mapping of TpBpy COF.

The TpBpy COF was synthesized via the Schiff-base condensation reaction between the electron-rich aromatic *C_3_*-symmetric Tp and *C_2_*-symmetric Bpy at room temperature, by using ionic liquids [BmimN(CN)_2_] as a catalyst. The obtained TpBpy COF was an aggregate of nanorods and presented an irregular geometric shape (Figure 1A). The elemental composition of TpBpy COF was assessed by energy dispersive X-ray spectroscopy (EDS), and Figure 1B–D showed that the elements (C, N, O) of TpBpy COF were distributed evenly.

**Figure 2:**
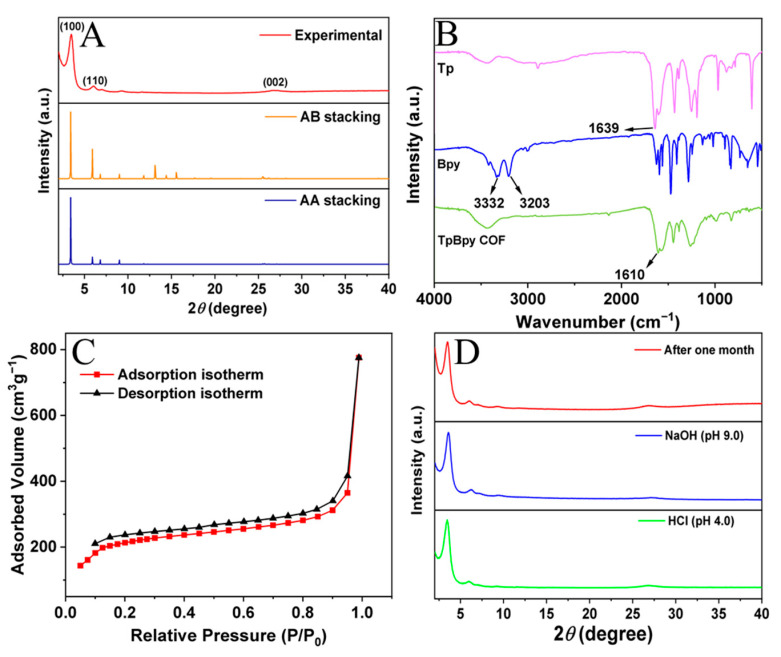
(**A**) Experimental and the simulated XRD pattern of TpBpy COF. (**B**) FT-IR spectra of Tp, Bpy, and TpBpy COF. (**C**) N_2_ adsorption and desorption isotherm of TpBpy COF. (**D**) XRD pattern of TpBpy COF after one month of storage and treatment in HCl (pH 4.0) and NaOH (pH 9.0).

Figure 2A showed the powder X-ray diffraction (PXRD) pattern of TpBpy COF. TpBpy COF showed an intense peak at *2θ* = 3.6°, and two weak peaks at *2θ* = 6.0° and 26.0°, corresponding to the (100), (110), and (002) planes [29], which were in good agreement with the simulated PXRD pattern by the AA stacking mode. The intense peak at 3.6° suggested the presence of open channels in the as-synthesized COF, which is conducive to fast electron transfer [30].

In the FT-IR spectra of Tp (Figure 2B), the presence of intense peak at ~1639 cm^−1^ of Tp was attributed to the asymmetric stretching vibration of -C=O. For Bpy, the bands at ~3203 cm^−1^ and ~3332 cm^−1^ represented the stretching vibrations of -N-H of aromatic amines. In the FT-IR of TpBpy COF, the -N-H stretching bands (~3203, ~3332 cm^−1^) and the -C=O stretching band (~1639 cm^−1^) disappeared due to the occurrence of Schiff-base condensation reactions, and a new characteristic peak appeared at ~1610 cm^−1^, which can be attributed to the formed imine bond -C=N [31].

The Brunauer–Emmett–Teller (BET) surface area of TpBpy COF was evaluated by N_2_ adsorption and desorption measurement at 77 K (Figure 2C). The surface area of TpBpy COF was deduced to be 874.1 m^2^ g^−1^, and the isotherm of TpBpy COF was accorded with type IV reversible adsorption isotherm [28,32], indicating the mesoporous structure of TpBpy COF. As shown in Figure 2D, the crystallinity of TpBpy COF was well maintained after one month of storage at room temperature. Compared with the pristine TpBpy COF, nearly no change was observed for the XRD pattern of the material after treatment with acid (pH 4.0 HCl) and base (pH 9.0 NaOH), indicating the excellent physical and chemical stability of the TpBpy COF.

### 3.2. Oxidase-Like Activity of TpBpy COF under Visible Light Illumination

The obtained TpBpy COF was mixed with three chromogenic substrates (TMB, ABTS, and OPD), and the resultant mixtures were irradiated with a white-light LED lamp (λ ≥ 420 nm) for 15 min to explore the oxidase-like activity of TpBpy COF. The corresponding UV-vis absorption spectra were recorded.

**Figure 3:**
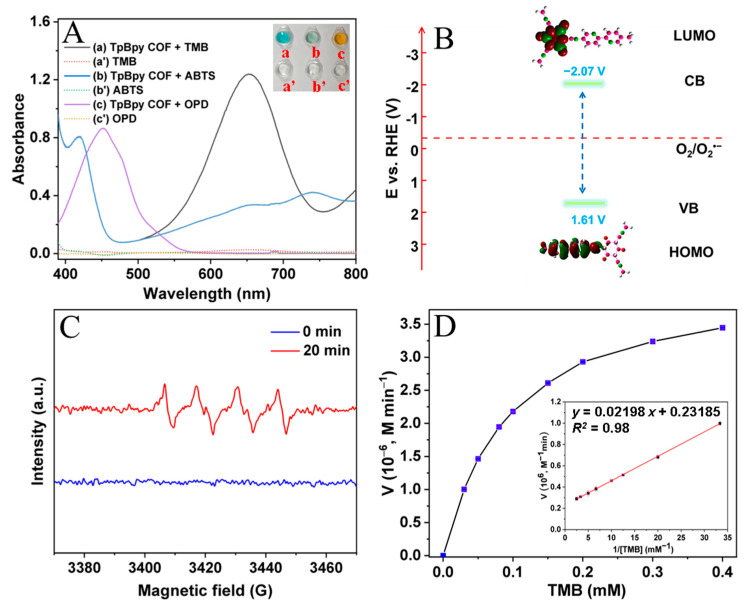
(**A**) UV-vis spectra of different chromogenic substrates with/without TpBpy COF in the HAc-NaAc buffer (0.2 mol L^−1^, pH 4.0). Insert: the images of the system containing TMB (a, a’), APTS (b, b’), and OPD (c, c’). (**B**) Density functional theory (DFT) calculation of TpBpy COF (Insert: the images of LUMO and HOMO). (**C**) EPR spectra of TpBpy COF after illumination under white-light LED lamp for 20 min and under dark environment. (**D**) Michaelis–Menten and Lineweaver–Burk curves of TpBpy COF against the different concentrations of TMB.

As depicted in Figure 3A, compared with a single TMB, a distinct absorbance peak at 652 nm was found for the TMB + TpBpy COF system, and an obvious color change from colorless to blue was observed with the addition of TpBpy COF. Similarly, ABTS and OPD changed from colorless to green and yellow, respectively, with the presence of TpBpy COF, and the corresponding absorption peak appeared at 410 nm and 445 nm for ABTS + TpBpy COF and OPD + TpBpy COF system, attributing to the produce of oxABTS and oxOPD. Therefore, all the above results suggested that the TpBpy COF had excellent oxidase-like activity under visible light irradiation.

Figure 3B shows the HOMO and LUMO levels of TpBpy COF. DFT calculation results revealed that the LUMO position of TpBpy COF (−2.07 V vs. Ag/AgCl) was much more negative than that of O_2_/O_2_^•−^ redox potential (−0.33 V vs. Ag/AgCl) [33], suggesting that the electrons in the LUMO of TpBpy COF could theoretically reduce O_2_ to generate O_2_^•−^, which then oxides the colorless TMB into blue oxTMB. In order to verify that the oxidation of TMB was caused by the O_2_^•−^ generated by TpBpy COF, the electron paramagnetic resonance (EPR) analysis was carried out to figure out the generation of O_2_^•−^ by using 5,5-dimethyl-1-pyrroline N-oxide (DMPO) as radical trappers. As shown in Figure 3C, the signal of O_2_^•−^ was clearly observed after light irradiation for 20 min, confirming that the generation of O_2_^•−^ was caused by photo-induced electron transfer of TpBpy COF.

To understand the kinetic mechanism of the oxidase-like activity of TpBpy COF, apparent steady-state kinetic parameters of the catalytic oxidation reaction are determined by changing the concentrations of TMB under fixed TpBpy COF levels. A series of initial reaction rates are calculated and applied to the double reciprocal of the Michaelis–Menten equation [29]:

*V = V_max_ [S]/(K_m_ + [S])*

where *[S]* was TMB content, *K_m_* resembles the Michaelis–Menten constant, *V* was the initial velocity of the reaction, and *V_ma_*_x_ was the maximal velocity of TMB oxidation.

As shown in Figure 3D, the absorbance increased gradually with TMB concentration. The results obtained from Lineweaver–Burk plots indicated that the Michaelis–Menten constant (*K_m_*) and the maximum initial velocity (*V_max_*) of TpBpy COF were deduced to be 94.8 μmol L^−1^ and 4.31 *×* 10^−6^ M min^−1^, respectively. Generally, K_m_ has been identified as an indicator of enzyme affinity with substrates. Compared with other oxidase-like catalysts (Table 2), the *K_m_* value of TpBpy COF was lower, suggesting the stronger affinity of TpBpy COF toward TMB than other oxidase-like catalysts.

### 3.3. Analytical Performance for Colorimetric UA Sensing

**Figure 4:**
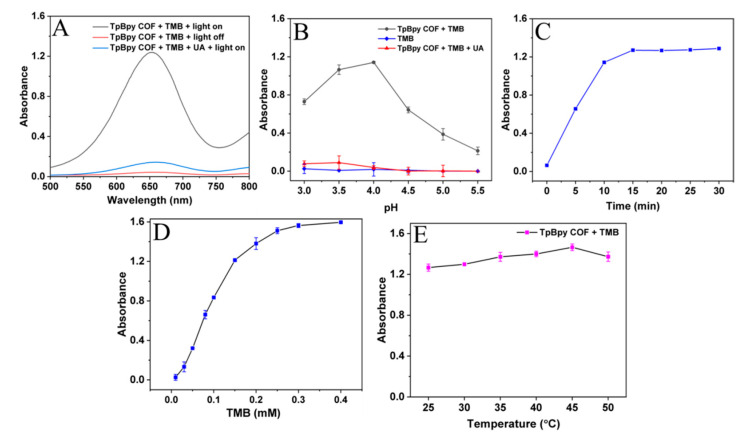
(**A**) UV-vis spectra of TpBpy COF + TMB system with/without the presence of UA. (**B**) The effect of pH on the absorbance at 652 nm of TpBpy COF + TMB, TpBpy COF, TMB, TpBpy COF + TMB + UA under visible light irradiation. The effect of irradiation time (**C**), TMB concentration (**D**), and temperature (**E**) on the absorbance at 652 nm of TpBpy COF + TMB.

The above investigations revealed that the TpBpy COF exhibited favorable oxidase-like activity and could oxide the colorless TMB into blue oxTMB. Meanwhile, it has been demonstrated that UA was able to reduce the blue oxTMB into colorless TMB [21,27]. Figure 4A showed the recorded UV-vis spectra of the TpBpy COF + TMB system before/after the addition of UA. It could be seen that the presence of UA induced an obvious decrease in the absorbance of the TpBpy COF + TMB system, and thus a colorimetric procedure was developed for UA sensing.

To achieve the best UA sensing performance, experimental parameters, including pH value, irradiation time, and TMB concentration, were optimized. It was found that TpBpy COF presented the highest oxidase-like activity under pH 4.0 (Figure 4B), and the absorbance difference of TMB + TpBpy COF system with/without UA reached the maximum at the same pH condition. The reason might be connected to the fact that TMB oxidation tends to form yellow diimine in excessive acid conditions and the decreased solubility of TMB under high pH conditions. Figure 4C illustrated the influence of irradiation time. The absorbance signal firstly increased obviously with the irradiation time, and, then, kept relatively stable when the irradiation time was longer than 15 min. The effect of TMB concentration was investigated in the range of 0.01–0.4 mmol L^−1^ (Figure 4D). The absorbance difference increased rapidly with the TMB content up to 0.25 mmol L^−1^, and then the signal increase became slow with the further increase in TMB concentrations. Figure 4E shows the influence of temperature. It is obvious that the increase in temperature just contributed slightly to the oxidase-like activity of TpBpy COF. For the comprehensive consideration of manipulation convenience and detection sensitivity, UA detection was carried out under a temperature of 25 °C in the present study.

Based on the comprehensive consideration of detection sensitivity and analytical cost, the colorimetric sensing procedure for UA detection was performed under pH 4.0, with an irradiation time of 15 min and a TMB concentration of 0.25 mmol L^−1^.

**Figure 5:**
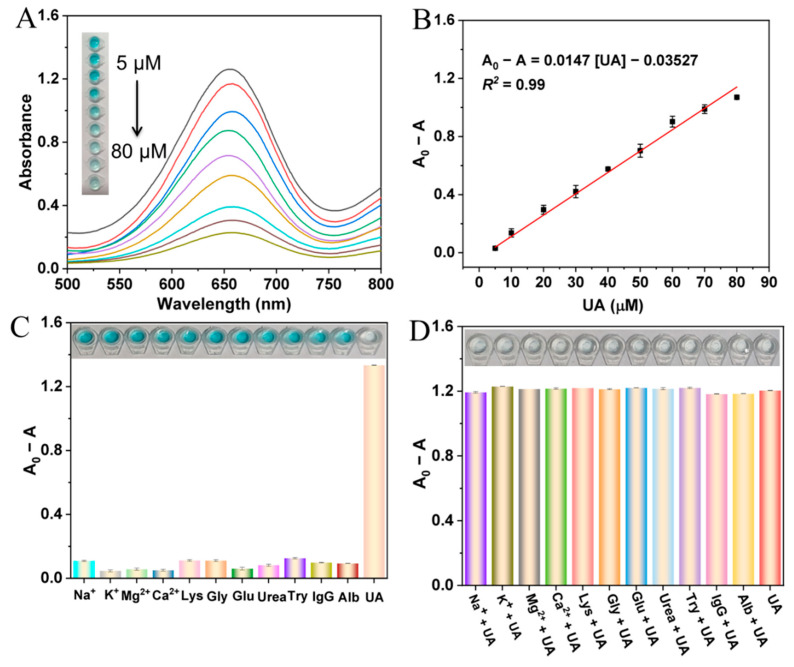
(**A**) UV-vis spectra of the TpBpy COF + TMB system toward different concentrations of UA (from up to down: 5, 10, 20, 30, 40, 50, 60, 70, and 80 μmol L^−1^) Insert: the images of the corresponding solutions. (**B**) The linear relationship between the absorbance difference (A_0_ − A) and UA content (A_0_ and A represented the absorbance at 652 nm in the absence/presence of UA). (**C**) The response of TpBpy COF + TMB system towards UA and various interferences. (**D**) The signal response of TpBpy COF + TMB system towards UA coexisting with other substrates. The concentration of UA was 100 μmol L^−1^, IgG and Alb was 1 mg L^−1^ and the concentration of other interferences was 1 mmol L^−1^.

Figure 5A illustrates the spectra of the TpBpy COF + TMB system with the addition of different concentrations of UA under optimal conditions. The absorbance difference (A_0_ − A) presented a good linear relationship with UA in the concentration range of 5–80 μmol L^−1^ (R^2^ = 0.98) (Figure 5B), and the limit of detection (LOD, 3σ/k) was deduced to be 1.7 μmol L^−1^. Table 3 summarized the performance of reported materials for UA sensing. It could be seen that TpBpy COF-based sensing system provided improved sensing sensitivity due to the excellent oxidase-like activity of TpBpy COF.

To evaluate the selectivity of the TpBpy COF-based system for UA sensing, the response of the sensing system was investigated in the presence of potentially interfering substances that coexisted in biological samples, including Na^+^, K^+^, Mg^2+^, Ca^2+^, lysine (Lys), glycine (Gly), tryptophan (Try), glucose (Glu), urea, albumin (Alb), and immunoglobulin (IgG). As illustrated in Figure 5C, only the UA induced obvious color fading and significant absorbance reduction in comparison with other substances. Moreover, those potential interfering substances nearly pose no influence on the signal of the sensing system when they coexisted with UA (Figure 5D). These demonstrated the favorable anti-interference ability of the TpBpy COF-based sensing system.

### 3.4. Smartphone Sensing Platform for UA Detection

**Figure 6:**
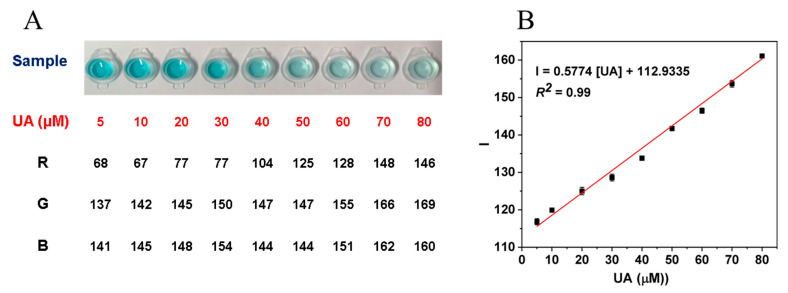
(**A**) Color changes in the TpBpy COF + TMB system and the corresponding RGB value with various content of UA (Red words represent different concentrations of UA). (**B**) The linear response of color intensity acquired by the RGB mode under different UA concentrations.

Due to the fact that the presence of UA could induce obvious color fading of the TpBpy COF-based sensing system, a smartphone-based platform was further developed for instrument-free and on-site detection of UA. The high-resolution photos of the sensing system with different UA concentrations under white-light irradiation were captured and converted into color intensity based on RGB mode by the “Color Grab” app. The YUV color space method with a weighted average of R, G, and B components recommended by the International Telecommunication Union (ITU) was adopted to construct luminance and color-difference signals [42], and the color intensity was calculated by using the following formula: I = 0.3 R + 0.59 G + 0.11 B [43]. As shown in Figure 6, the calculated RGB value “I” exhibited a good linear relationship in the UA concentration range of 5–80 μmol L^−1^ (R^2^ = 0.99), giving access to the UA detection by a portable smartphone independent of lab equipment, and the detection limit was 3.1 μmol L^−1^.

### 3.5. Determination of UA Content in Human Serum and Urine Samples

To verify the practicability of this TpBpy COF-based sensing system, UA content in human urine and serum samples were determined via the colorimetric procedure and the smartphone-based platform.

As shown in Table 4, the spiked recoveries for UA in urine samples and serum samples were 97.2–107.8% and 96.6–100.2%, respectively. Meanwhile, the UA contents in the biological samples that determined the TpBpy COF-based colorimetric procedure were in good agreement with that obtained by means of a smartphone-based platform. All these results revealed that the TpBpy COF-based sensor owns great potential in the detection of UA contents in practical applications.

## 4. Conclusions

In this study, we successfully synthesized a two-dimensional covalent organic framework TpBpy COF via the Schiff-base condensation reaction. The as-synthesized TpBpy COF exhibited excellent oxidase-like activity under visible light irradiation that could efficiently catalyze the TMB into blue oxTMB. Based on the ability of UA to reduce oxTMB, we proposed two modes (batch colorimetric procedure and smartphone signal readout platform) for UA sensing. The UA contents determined by the smartphone-based signal readout platform are consistent with the theoretic UA contents in healthy human serum and urine [2], suggesting that the TpBpy COF-based smartphone signal readout system is able to act as a powerful platform for point-of-care UA content evaluation free of sophisticated instruments, which might provide useful and timely information for preliminary screening of UA-related diseases.

## Figures and Tables

**Table 1:** Comparisons of different methods for UA detection.

Method	Sensitivity	Selectivity	Rapidity	Precision	Complex Sample Preparation Process	Expensive Instruments	Low Cost
Colorimetry	√	√	√	х	х	х	√
HPLC	√	√	√	√	√	√	х
Fluorescence	√	√	√	√	х	√	√
Chemiluminescence	√	х	√	√	√	х	√
Electrochemistry	√	√	√	√	√	х	√

**Table 2:** The kinetic parameters of oxidation reactions catalyzed by reported enzyme-like nanomaterials and horseradish peroxidase.

Catalyst	*K_m_* (mM)	*V_m_* (10^−6^ M min^−1^)	Ref.
TpBpy COF	0.0948	4.31 × 10^−6^	This work
PtNPs@ZIF	0.15	7.26 × 10^−6^	[18]
CuSNPs	0.216	1.45 × 10^−5^	[34]
Fe_3_O_4_@MIL-100 (Fe)	0.112	6.85 × 10^−6^	[35]
HRP	0.4340	6.00 × 10^−6^	[23]

**Table 3:** The analytical performance of reported materials for UA sensing.

Material	Linear Range (μM)	LOD (μM)	Ref.
TpBpy COF	5–80	1.7	This work
Uricase/graphitic carbon nitride	10–100	8.9	[36]
Ni glancing angle deposition film	0–6	3.3	[37]
Lanthanide-doped upconversion nanoparticles	10–1000	2.86	[38]
Au/Ag nanoclusters	5–50	5.1	[39]
TCPO-H_2_O_2_-rubrene	10–1000	5	[40]
Photochemically reduced graphene oxide	40–415	8	[41]

**Table 4:** Determination of UA content in human urine and serum samples (*n* = 3).

Sample	Added (μM)	Colorimetric Determination (μM)	Recovery (%)	RSD (%, *n* = 3)	Smartphone Determination (μM)	Recovery (%)	RSD (%, *n* = 3)
Urine	0	24.3 ± 1.2		4.8	23.3 ± 0.8		3.3
	10.0	34.7 ± 2.0	103.6 ± 2.0	5.6	34.1 ± 0.6	107.1 ± 5.6	1.6
	30.0	55.0 ± 1.1	102.3 ± 3.5	1.9	55.7 ± 0.6	107.8 ± 2.0	1.1
	50.0	73.0 ± 0.7	97.2 ± 1.3	0.9	73.8 ± 1.6	100.8 ± 3.2	2.3
Serum	0	4.5 ± 0.2		3.6	4.6 ± 0.1		2.4
	10.0	14.3 ± 0.4	98.8 ± 3.5	2.7	14.3 ± 0.9	97.1 ± 0.8	5.9
	30.0	33.9 ± 0.4	98.1 ± 1.4	1.3	33.6 ± 0.4	96.6 ± 1.4	1.2
	50.0	54.5 ± 0.4	100.1 ± 0.9	0.7	54.7 ± 0.8	100.2 ± 1.6	1.4

## Data Availability

The data presented in this work are available in the article.
